# Effects of Esketamine on Acute and Chronic Pain After Thoracoscopy Pulmonary Surgery Under General Anesthesia: A Multicenter-Prospective, Randomized, Double-Blind, and Controlled Trial

**DOI:** 10.3389/fmed.2021.693594

**Published:** 2021-09-08

**Authors:** Yishan Lei, Huayue Liu, Fan Xia, Shulin Gan, Yulan Wang, Wenwen Huo, Qinyun Wang, Fuhai Ji

**Affiliations:** Department of Anesthesiology, The First Affiliated Hospital of Soochow University, Suzhou, China

**Keywords:** esketamine, acute pain, chronic pain, post-thoracotomy pain syndrome, thoracoscopy pulmonary surgery

## Abstract

**Background:** Post-operative pain management for patients undergoing thoracoscopy surgery is challenging for clinicians which increase both health and economic burden. The non-selective NMDA receptor antagonist esketamine possesses an analgesic effect twice that of ketamine. The application of esketamine might be beneficial in alleviating acute and chronic pain after thoracic surgery. The current study describes the protocol aiming to evaluate the analgesic effect of esketamine after pulmonary surgery via visual analog scale (VAS) score for acute and chronic pain.

**Methods:** A multi-center, prospective, randomized, controlled, double-blind study is designed to explore the analgesic effect of esketamine in randomized patients undergoing video-assisted thoracoscopic surgery (VATS) with general anesthesia. Patients will be randomly assigned to Esketamine Group (Group K) and Control Group (Group C) in a ratio of 1:1. Group K patients will receive esketamine with a bolus of 0.1 mg/kg after anesthesia induction, 0.1 mg/kg/h throughout the operation and 0.015 mg/kg/h in PCIA after surgery while Group C patients will receive the same volume of normal saline. The primary outcome is to measure the pain intensity through the VAS score at 3 months after the operation. The secondary outcome includes VAS score at 1, 4, 8, 24, and 48 h and on the 7th day and 1 month after the operation, complications, ketamine-related neurological side effects, recovery time of bowel function, and total amount of supplemental analgesics.

**Discussion:** The results of the current study might illustrate the analgesic effect of esketamine for patients undergoing thoracoscopy pulmonary surgery and provide evidence and insight for perioperative pain management.

**Study Registration:** The trial was registered with Chinese Clinical Trial Registry (CHICTR) on Nov 18th, 2020 (ChiCTR2000040012).

## Introduction

Despite of the wide spread of minimally invasive video-assisted thoracoscopic surgery (VATS), post-operative pain after thoracotomy is still a challenge for anesthesiologists (1, 2). Thoracic epidural analgesia, paravertebral block, intercostal nerve block, as well as multimodal analgesia ameliorate acute post-operative pain in patients undergoing thoracic surgery (3), nonetheless the transition from acute post-operative pain to chronic post-thoracotomy pain syndrome (PTPS) is a common issue, which might lead to sputum clearance impairment and hypopnea thus resulting in increasing health and economic burden. As reported in previous studies, the incidence of chronic pain after thoracotomy at 3- and 6-months ranges from 47 to 80% (4, 5). The International Association for the Study of Pain (IASP) defines PTPS as “recurring or persistent pain along an open chest incision at least 2 months after surgery” (6). PTPS is often described as burning, localized and discrete tenderness, and persistent paresthesia around the incision site, which might ascribe to the complex interaction of peripheral and central sensitization. Previous studies demonstrated that proper treatment for acute pain might relate to lower incidence and intensity of chronic pain (7). Besides, under-controlled acute pain within 1–3 days after the VATS might result in transition to chronic pain, which lasts for 6 months or even longer.

Non-opioids and analgesic interventions that aim to reduce opioids consumption for patients undergoing thoracotomy improve recovery and alleviate opioid-related side effects. Ketamine, a non-selective NMDA receptor inhibitor, possesses part of non-opioid analgesic properties. Studies have shown that low-dose intravenous infusion of ketamine can be used as an adjuvant drug to acute and chronic post-operative pain management (8–13). Intravenous administration of opioids for post-operative analgesia might cause hyperalgesia and tolerance to opioids, both of which are partially related to NMDA receptor activation. Preventive and post-operative analgesia with NMDA receptor antagonists can prevent acute tolerance of opioids and reduce the development of neuropathic pain (14). Despite these advantages, the side effects of ketamine including nightmares and delusions, refine its routine use (15, 16).

Esketamine, the S (+)-isomer of ketamine, has an analgesic effect twice that of racemic ketamine. Esketamine possesses the advantages of a lower incidence of side effects like hallucinations, faster recovery, and the ability to lower MAC value of sevoflurane as well as protect hypoxic pulmonary vasoconstriction during one-lung ventilation (17). Studies indicated that esketamine might alleviate PTPS, which might ascribe to prevention of central sensitization (18). Theoretically, esketamine might be promising in perioperative pain management, yet it remains elusive that whether it is advantageous in the treatment of acute and chronic pain after thoracoscopic lung surgery. In this issue, we design a multicenter-perspective, double-blind, randomized, and controlled study to investigate the analgesic effect of esketamine during perioperative timeline in patients undergoing VATS.

## Methods and Analysis

### Trial Objectives

**Figure 1:**
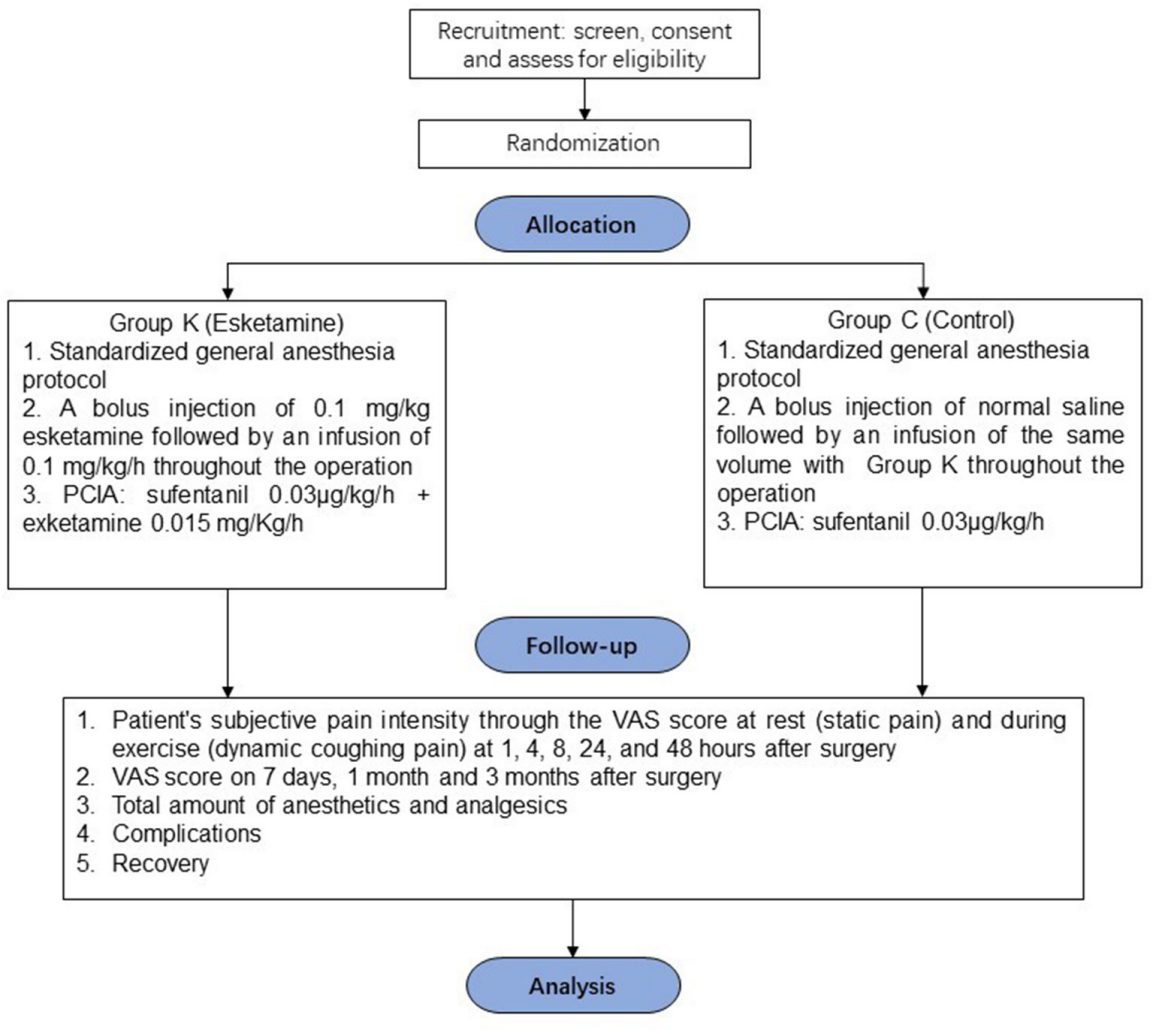
General review of study design.

To explore the analgesic effect of intravenous esketamine for acute and chronic pain in VATS patients, a multi-center, prospective, randomized, controlled, double-blind designed study will be launched to evaluate pain in randomized patients undergoing VATS with general anesthesia by a visual analog scale (VAS) score in the time points within 48 h and on the 7th day, 1 and 3 months after the operation. The results of this study might provide new insights for pain management in patients undergoing VATS. The flow of participants through the trial is summarized in Figure 1.

### Participants-Inclusion and Exclusion Criteria

#### Inclusion Criteria

1. Age 18–65 years old;
2. ASA I~III level;
3. Plan to perform general anesthesia with tracheal intubation for elective VATS including: lung wedge resection, lung segment resection, partial lung resection, lobectomy, and radical resection of unilateral lung cancer;
4. Plan to be hospitalized ≥48 h after surgery;
5. Be willing to use patient controlled intravenous analgesia (PCIA).

#### Exclusion Criteria

1. ASA >III level;
2. History of allergy to either anesthetics, ropivacaine, or ketamine; Allergy to or have any contra-indications to non-steroidal anti-inflammatory drugs (NSAIDs);
3. Mental illness and chronic pain that may confuse analgesic effect;
4. Cannot read and write Chinese, communication difficulty;
5. Emergency surgery or trauma patients;
6. Have a history of intraoperative awareness;
7. Transfer to ICU after surgery;
8. Unwilling to use PCIA or refuse to participate in this study.

#### Drop Out Criteria

1. Reoperation during the observation period;
2. Unconsciousness or mortality during the observation period;
3. Discharge automatically or transferred in advance;
4. The patient or the client refuses the informed consent or requests to withdraw from the study during the observation period;
5. Interruption of PCIA due to severe nausea and vomiting.

### Trial Groups and Procedures

#### Trial Groups

Patients will be divided into two groups according to a computer-generated randomized number table in a ratio of 1:1: Group K (esketamine group) and Group C (control group).

In group K (*n* = 457), patients receive a bolus injection of 0.1 mg/kg esketamine intravenously after anesthesia induction, followed by a continuous intravenous infusion of 0.1 mg/kg/h throughout the operation. Patients will be equipped with a PCIA (sufentanil 0.03 μg/kg/h + esketamine 0.015 mg/Kg/h) in a total volume of 100 ml and continuous infusion at a rate of 1.5 mL/h for 48 h. The self-controlled capacity is 1.5 ml, and the locking time is 10 min.

In group C (*n* = 457), patients received an injection of normal saline after anesthesia induction and followed by a continuous intravenous infusion of normal saline with the same volume as group K. After the operation, a PCIA will be used (sufentanil 0.03 μg/kg/h only, total volume 100 ml, 1.5 mL/h for 48 h). The self-controlled capacity is 1.5 ml, and the locking time is 10 min.

#### Procedures

All intraoperative and post-operative procedures have been standardized as follows.

### Monitoring and Anesthesia

Monitoring includes radial artery blood pressure, electrocardiogram, pulse oxygen saturation, respiration, end-tidal carbon dioxide concentration and BIS. For general anesthesia induction, midazolam 1–3 mg, sufentanil 0.3–0.6 μg/kg, Propofol 2~3 mg/kg, and cisatracurium 0.2 mg/kg will be infused intravenously followed by double-lumen bronchial intubation according to surgical needs.

For anesthesia maintenance, intermittent or continuous intravenous bolus of cisatracurium 0.05~0.1 mg/kg and inhalation of sevoflurane 1–3% or continuous pumping of propofol will be used to maintain BIS value between 40 and 60. The total amount of sufentanil during the surgery is 0.4–1 μg/kg, and 0.1–0.2 μg/kg will be added intermittently as needed. At the beginning and end of the operation, NSAIDs (such as flurbiprofen axetil 50 mg) will be applied intravenously, and antiemetic drugs (such as ondansetron 8 mg) will be injected intravenously at the end of the operation. Use antiemetics on the first and second days post-operatively. During the operation, the systolic blood pressure and heart rate will maintain within 20% of the baseline.

Before the skin incision, the anesthesiologist or surgeon will perform local infiltration anesthesia with 0.375% ropivacaine 5 ml in each incision. The number of incisions depends on the needs of the operation. An additional local infiltration of 0.375% ropivacaine 5 ml will be carried out around the drainage tube incision when requiring thoracic drainage.

One lung ventilation on the contralateral lung will be performed once skin incision starts. The tidal volume is set to 6–8 mL/kg of the ideal body weight, with respiratory frequency 12–16 breaths/min, and the oxygen concentration is up to 100% as needed. Set the PEEP as 3–5 cmH_2_O.

### Post-operative Analgesia

PCIA will be applied after the operation with continuous infusion at a rate of 1.5 mL/h for 48 h. If VAS >5, 1 mg morphine will be injected intravenously in addition to full use of PCIA. Besides, change the PCIA setting to a capacity of 2 ml for self-control and 5 min for the locking time. Patients can orally take acetaminophen (2 g/day) or tramadol (300 mg/day) or other drugs as supplemental analgesics. The type and dose of the drug should be recorded.

If the VAS is found to be continuously lower than 1, and either respiratory depression, confusion, and unstable blood pressure occurs, the PCIA infusion should be suspended. Use of PCIA should be restarted until the above-mentioned manifestations improves.

### Evaluation and Follow-Up

Each patient will be given a VAS form to record score of the most severe pain, the painful location, and the total amount of analgesics within 3 months after surgery. Mild PTPS is defined as VAS between 0 and 3 that does not affect daily activities, and a VAS between 4 and 6 that has hindered normal quality of life is defined as moderate PTPS.

Researchers who are unaware of the research intervention will collect all patient data and follow up, including the following: gender, age, weight (kg), body mass index (kg/m2), FEV1%, operation time (minutes), anesthesia time (minutes), days of hospitalization, days of chest drainage, post-operative evaluation, complications, side effects (respiratory depression, hypotension, vomiting, nausea, itching, and ketamine related neurological side effects), total supplemental analgesics within 48 h after surgery, recovery time of bowel function, and length of hospital stay (days) after surgery. Vital signs are measured every hour from the surgery to 24 h after the surgery, and then every 4 h from 24 to 48 h after the surgery.

### Adverse Events

1) Tachycardia: When heart rate >100 beats/min, or increases by more than 20% from baseline if the baseline value is >83 beats/min, esmolol 10 mg will be given and/or adjust the dose of anesthetics;
2) Hypertension: systolic blood pressure >160 mmHg, or increases from baseline 20% or more if the baseline value >133 mmHg, nicardipine 0.5 mg will be given and/or adjust the dose of anesthetics;
3) Bradycardia: Heart rate <55 beats/min, or reduces by more than 20% from baseline or if the baseline value is <69 beats/min, atropine 0.3 mg and/or isoproterenol 2 μg or adjust the anesthetics dose;
4) Hypotension: systolic blood pressure <95 mmHg, or drops more than 20% if the baseline value is <119 mmHg, liquid infusion, ephedrine 6 mg or norepinephrine 4 μg and/or anesthetics dose adjustment will be applied;
5) Intraoperative awareness: During general anesthesia and standard treatment, patients can recall intraoperative events.

Any adverse event should be recorded including type, time, duration, treatment, and sequelae and follow up until it is completely resolved or treatment is terminated. A serious adverse event is defined as any serious medical event that causes death, life-threatening, prolonged hospital stay, persistent disability or dysfunction, or other unpredictable serious medical events. If there are any serious adverse events that might cause mortality, be life-threatening, prolong hospital stay or result in persistent disability or dysfunction, the study will be terminated and treatment should start immediately.

### Data Collection, Handling, and Monitoring

Preoperative, intraoperative and follow-up data will be recorded by researchers who are unaware of the research intervention as listed in the following (Table 1).

**Table 1:** Data collection throughout the trial.

**Baseline data**
1. General questions including age, gender, race, education, occupation, live alone or not, height, weight, and BMI
2. Hospitalization information including admission date, preoperative diagnosis, and VATS type planning
3. Medical history: smoking and alcohol history, history of lung surgery, cardiovascular diseases, chronic pain, and preoperative medication
4. Auxiliary examination including ECG, X-ray or CT, echocardiography, serum sodium, serum potassium, serum glucose, serum BNP, serum urea nitrogen, serum albumin, serum CRP, serum creatine, hemoglobin, and Hct
**Intraoperative data**
1. Surgery information: surgery date, duration, type, incision number, and drainage tube number
2. Vital signs: heart rate and MAP before anesthesia induction, duration of heart rate >100/min and <40/min, duration of MAP >120%, 130%, and <70%, 80% of the baseline, allergy, and cardiac arrest
3. Anesthesia information: duration, total anesthetics amount (propofol, sufentanil, cisatracurium, NSAIDs), dose of vasoactive drugs and capacity parameters (blood loss, liquid infusion, and urine volume)
**Post-operative data**
1. Vital signs: Blood pressure, heart rate, SpO_2_ in 1, 4, 8, 24, and 48 h after surgery
2. VAS score: static and dynamic coughing pain in 1, 4, 8, 24, and 48 h after surgery, pain located in incision and drainage tube on 7 days, 1 month, and 3 months after surgery
3. Analgesics: total amount of analgesic (including morphine, acetaminophen, etc.) within 3 months after surgery
4. Pain and daily activities: whether pain limits daily activities within 3 months after surgery
5. Others: duration for thoracic drainage, further treatment including radiotherapy and chemotherapy, mortality
**Post-operative complications**
1. General complications: intraoperative awareness, delayed recovery, stroke, pulmonary infection, incision infection, recovery time of bowel function, re-operation, and length of hospital stay
2. Amount of PCIA and related complications: total amount of PCIA and frequency of self-control within 48 h after surgery, complications including itch, sleepiness, nausea, and vomiting
3. Esketamine related complications: dreaminess, nightmare, illusion, delirium, restlessness, orientation difficulty, laryngospasm, blurred vision, tachycardia, and salivation within 48 h
4. Incision related complications: burning sensation, stabbing pain, sharp pain, itch, and heaviness within 3 months

### Outcome Measurements

#### Primary Outcome

The primary outcome of this study is to assess the patient's subjective pain intensity through the VAS score at 3 months after surgery. VAS score higher than 1 will be considered positive.

#### Secondary Outcome

The secondary outcome includes VAS score at 1, 4, 8, 24, and 48 h and on 7 days and 1 month after surgery, respiratory depression, hypotension, vomiting, nausea, itching, ketamine-related neurological side effects (including dreaminess, nightmare, illusion, delirium, restlessness, orientation difficulty, laryngospasm, blurred vision, tachycardia, and salivation), and recovery time of bowel function. Besides, the total amount of supplemental analgesics within 48 h and 3 months after surgery will also be documented.

### Randomization, Blinding, Allocation, and Concealment

The study is a multi-center, prospective, double-blind, randomized, parallel controlled trial. The only person who knows which group the patients are assigned to is the research coordinator, and the anesthesiologist in charge is not informed.

1. A statistician who is not involved in data management and statistical analysis possesses the randomized code list generated by computer. The ratio of numbers for Group K and Group C is 1:1;
2. The results of randomization will be sealed in sequentially numbered envelopes, stored and managed by the research coordinator;
3. The research coordinator is responsible for opening the randomization envelope, distributing experimental drugs, collecting data during the operation, and coordinating research. Anesthesiologists use the distributed experimental drugs to execute research programs;
4. The other two researchers will be responsible for the post-operative follow-up, who are blind to the randomized allocation of patients;
5. The anesthesiologists who carry out the research plan and the researchers who collect post-operative results data do not share any information related to patient randomization;
6. The statistical analysis will be carried out independently by a separately appointed statistician.

### Study Sample Calculation

The literature reports that the incidence of chronic pain after thoracotomy at 3 and 6 months after surgery ranges from 47 to 80%. We assume that the incidence of chronic pain after thoracotomy in this study is about 50% at 3 months after thoracotomy in patients in the current study, and that the incidence of chronic pain after thoracoscopy will be reduced by 1/5 in the esketamine group. We calculate the required sample size with PASS software with an alpha error of 0.05 and power 80% (test for two proportions, two-sided *T*-test) and figure out that each group include 388 patients. Considering ~15% dropout rate, 457 patients per group for a total of 914 patients will be recruited in this study.

### Data Management and Statistical Analysis

**Figure 2:**
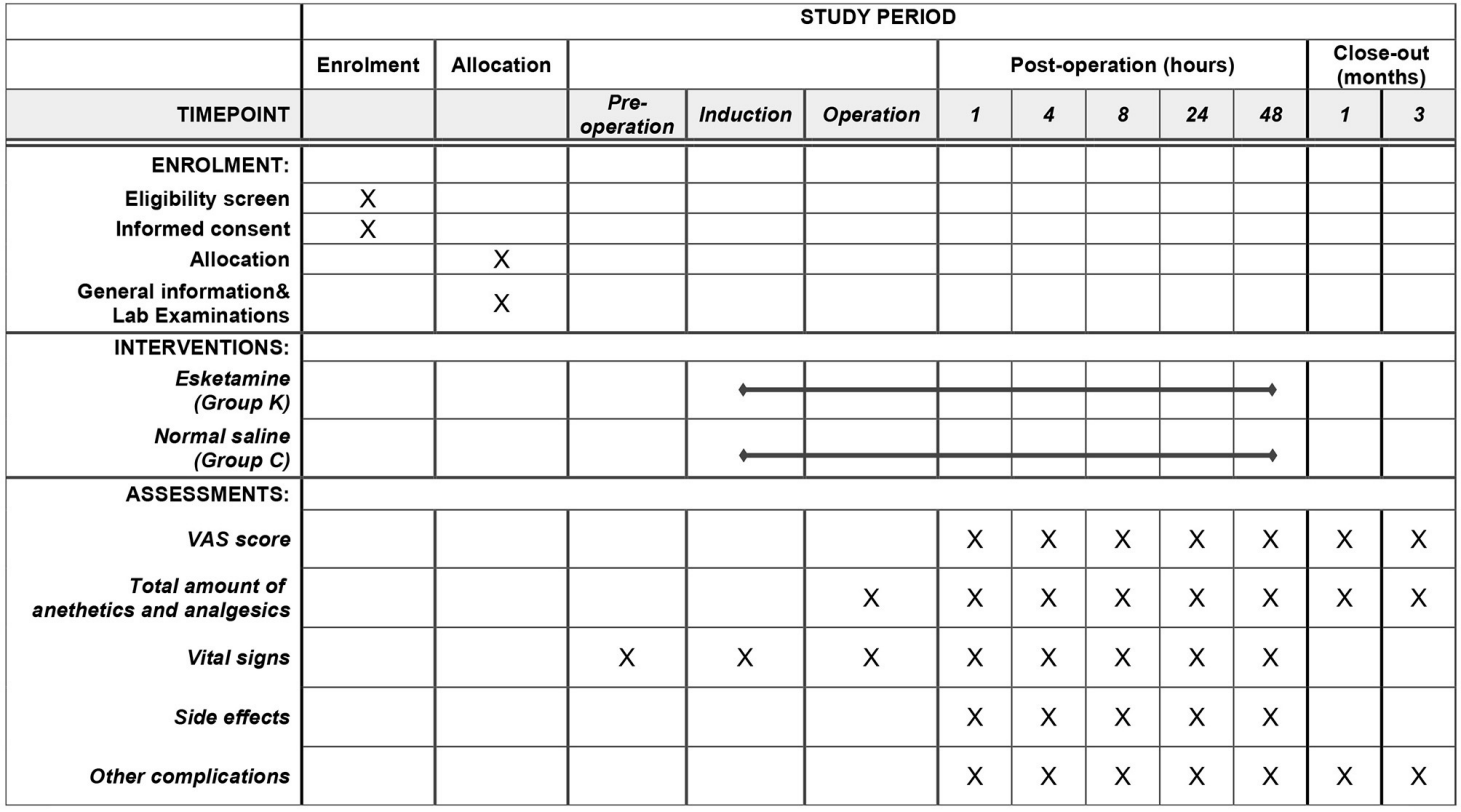
Study timeline and schedule of enrolment, allocation, interventions, and assessments according to SPIRIT 2013 statement.

The two-sided test will be used for all statistical analysis, and the *p* < 0.05 is statistically significant. *T*-test or Wilcoxon test is used to compare baseline numerical variables and time variables between groups. Chi-square test or Fisher's test is used compare categorical variables between groups. Repeated measure analysis of variance and Mann-Whitney test are used to compare post-operative VAS scores between groups. A planned interim analysis after half of the total patients recruited will be performed by an independent statistician. Interim analysis provides a possibility for sample size recalculation and early stop for the current study. An overview of the study protocol complies with the Standard Protocol Items: Recommendations for Interventional Trials (SPIRIT) guidelines is presented in Figure 2.

### Trial Registration

The trial was registered with Chinese Clinical Trial Registry (CHICTR) on Nov 18th, 2020 (ChiCTR2000040012). We started recruit participants after receiving the registration number.

## Discussion

To our best knowledge, the analgesic effect and clinical value of esketamine in patients undergoing lung surgery remains elusive. To explore this issue, we design a multi-center, prospective, randomized, controlled, double-blind study, which aims to illustrate the hypothesis that perioperative application of esketamine alleviates acute and chronic pain in patients undergoing VATS and reduces the incidence of PTPS.

As the S(+)-isomer of ketamine, esketamine possesses a mighty analgesic effect in theory. Nonetheless, the practical analgesic effect of esketamine still remains controversial. Previous studies have demonstrated that esketamine 0.5 mg/kg as a pre-incisional bolus followed by a continuous infusion 400 μg/kg/h reduced pain scores within 48 h after thoracotomy and reduced morphine consumption (19). In a chronic opioid-dependent population, perioperative esketamine bolus 0.5 mg/kg followed by 0.25 mg/kg/h infusion reduced pain and improve labor market attachment 1 year after spine surgery (20). During laparoscopic cholecystectomy under TCI anesthesia, continuous esketamine at a rate of 0.3 mg/kg/h infusion alleviated post-operative pain and reduced morphine requirement (21). In the contrary, a few studies showed that esketamine might have less effect in analgesia. In patients undergoing major open gynecological surgery, esketamine 0.25 mg/kg before skin incision and after uterus removal did not reduce the total morphine consumption (22). For patients undergoing total knee arthroplasty, pregabalin, and esketamine reduced piritramide consumption during the first 24 h post-operatively but increase side effects including diplopia and dizziness (23). In a randomized, double-blind study, esketamine had no effects in the prevention of PTPS at 3 and 6 months (24). In consideration of the disputable effect of esketamine, a multi-center, prospective, randomized, controlled, double-blind study focusing on the analgesic effect of esketamine on PTPS is of urgency.

Even though the application of multimodal analgesia for VATS patients, PTPS is still a formidable challenge for clinicians as its prevalence generally up to 47–80% with a duration of 6 months or longer. The high incidence of PTPS might partly ascribe to its elusive mechanism. According to neuropathic symptoms complained by patients and that nerve damage might accompany thoracic surgeries, neuropathic pain might be possible mechanism of PTPS which contains complex interaction of central and peripheral insults (25). Previous studies have demonstrated that glutamate, an excitatory neurotransmitter and its receptor contributes to the formation of neuropathic pain and might be a potential therapeutic target (26, 27). In case, the non-selective NMDA receptor antagonist ketamine might possess the analgesic capacity for PTPS. However, studies showed that ketamine alleviates acute pain for patients receiving thoracic surgery and a few studies showed no benefits. Furthermore, studies did not show evidence that ketamine might provide precaution in PTPS (28–32). In this background, we aim to explore the analgesic effect of intravenous esketamine for acute and chronic pain in VATS patients. The results of the current study might provide evidence and insight for peri-operative pain management.

## Ethics Statement

The studies involving human participants were reviewed and approved by Chinese Clinical Trial Registry. The patients/participants provided their written informed consent to participate in this study.

## Author Contributions

YL, HL, and FX designed the protocol and completed the manuscript. SG, YW, and WH contributed to the study design, registration and table, and figure preparation. FJ and QW designed the study and modified the manuscript. All authors contributed to the article and approved the submitted version.

## Funding

This study will be supported by Youth Science and Sanitary Foundation of Suzhou (kjxw2018005) and National Natural Science Foundation of China (81873925).

## Conflict of Interest

The authors declare that the research was conducted in the absence of any commercial or financial relationships that could be construed as a potential conflict of interest.

## Publisher's Note

All claims expressed in this article are solely those of the authors and do not necessarily represent those of their affiliated organizations, or those of the publisher, the editors and the reviewers. Any product that may be evaluated in this article, or claim that may be made by its manufacturer, is not guaranteed or endorsed by the publisher.
